# Effect of intensive care unit diary on quality of life of intensive care unit survivors and their relatives: A systematic review and meta‐analysis

**DOI:** 10.1002/nop2.1819

**Published:** 2023-05-31

**Authors:** Donghui Hu, Xiaoli Ji, Yaxin Li, YueNing Liang, Jia Chen

**Affiliations:** ^1^ School of Nursing Yanbian University Yanji China; ^2^ Yanbian Hospital Yanji China

**Keywords:** diary, intensive care unit, meta‐analysis, quality of life

## Abstract

**Aim:**

To evaluate the current literature on the effect of ICU diaries on the quality of life of ICU survivors and their relatives.

**Design:**

Systematic review and meta‐analysis.

**Methods:**

We searched the online databases Web of Science, PubMed, Embase, The Cochrane Library, CNKI and WanFang from inception to April 2021. Cochrane Risk of Bias Assessment Tool and Newcastle–Ottawa Scale (NOS) were used to assess the methodological quality.

**Results:**

Seven studies were identified. ICU diaries improved the QoL of ICU survivors (SMD 0.79, 95% CI 0.24–1.34), and a small study described no benefit of diaries in improving the QoL of relatives.

**Conclusion:**

This meta‐analysis revealed the use of ICU diaries to improve the QoL in survivors, but the ICU diaries do not have a beneficial effect on the QoL in relatives. The evidence summarized in our study is limited and biased, and more research should be carried out to verify it.

## 1 INTRODUCTION

In earlier critical care, patients' survival (understood as being discharged alive from the intensive care unit [ICU]) was a measure of success (Ridley, 2002). The rising prevalence of critical illness combined with advances in critical care medicine has resulted in an increasing number of patients (~80–90%) surviving to hospital discharge (Hill et al., 2016; Iwashyna et al., 2012; Prescott & Angus, 2018), and this number is expected to rise with the COVID‐19 pandemic (WHO, 2020). Now, with increased interest in the long‐term health outcomes of critical care survivors, the goal of critical care has shifted from survival to ‘moving on’ to life after critical illness (Kean et al., 2017).

However, ICU survivors may develop new or worsening impairments in physical, cognitive or mental impairment after ICU discharge, as may their family caregivers, which is summarized as Post‐Intensive Care Syndrome (PICS) or PICS‐Family (PICS‐F) (Needham et al., 2012). To the best of our knowledge, not all patients develop PICS and the prevalence of PICS ranges from 6% to 57% (Connolly et al., 2016). Physical (ICU‐AW), cognition (delirium, memory loss) and mental health (depression, anxiety and PTSD) impairments in PICS can last for a few months to many years (Needham et al., 2012), leading to increased healthcare costs, re‐hospitalization, joblessness, increased use of primary care and a decrease in health‐related quality of life (Davydow et al., 2014; Kamdar et al., 2017; Naaktgeboren et al., 2022; Norman et al., 2016; van Beusekom et al., 2019; Wintermann et al., 2019).

Recently, with the increase in PICS‐related research, several interventions have been proposed to prevent and reduce PICS, such as the ABCDE bundle, ICU diary, post‐ICU recovery clinic and early rehabilitation. The diary is widely used as a tool for ICU patients to fill in memory gaps and help them recover (Olsen et al., 2017). This helps patients to correctly understand traumatic events through exposure to traumatic memories, thus reducing their associated symptoms (Locke et al., 2016). ICU diaries are now a non‐invasive and low‐cost ICU intervention (Sayde et al., 2020) that has been promoted for use on critically ill patients or their relatives (Garrouste‐Orgeas et al., 2017).

More research on ICU diaries is conducted and more meta‐analyses are published. However, in most studies, the effectiveness of ICU diaries was measured primarily in PTSD, anxiety and depression (McIlroy et al., 2019; Nydahl et al., 2019; Sun et al., 2021). Two earlier observational cohort designs and recent randomized controlled studies evaluated the impact of ICU diaries on QoL (Bäckman et al., 2010; Nielsen et al., 2019; Svenningsen et al., 2014), yet reported differing opinions. Two recent meta‐analyses summarized these findings suggesting a benefit of ICU diaries (Barreto et al., 2019; McIlroy et al., 2019). However, which is limited by observational studies and the quantity of studies included. Furthermore, few studies report the efficacy of ICU diaries on the QoL of relatives. Since the two meta‐analysis publications, three randomized controlled trials were published (Liu et al., 2019; Wang, 2017; Wang et al., 2020).

Therefore, we aimed to re‐evaluate the current research and update the effect of ICU diaries compared to no diaries in improving the QoL of ICU survivors and their relatives.

## 2 THE REVIEW

### 2.1 Aim

The purpose of this systematic review and meta‐analysis was to evaluate current research on the effect of ICU diaries on the QoL of ICU survivors and their relatives, as well as to analyse the effect on the domains of QoL.

### 2.2 Design

This systematic review and meta‐analysis was conducted and reported according to the PRISMA guidelines and registered in the PROSPERO (CRD42021251916).

### 2.3 Ethics

The data used in this systematic review and meta‐analysis were obtained from previously published studies, and therefore, this systematic review and meta‐analysis did not require ethical approval.

### 2.4 Search methods

We searched the online databases Web of Science, PubMed, Embase, The Cochrane Library, China National Knowledge Infrastructure (CNKI) and WanFang from inception to April 2021 using Mesh and Entry terms, aiming to capture those publications which contained variations of ‘ICU survivor,’ ‘diary/⽇记,’ ‘Intensive Care Units/ICU/重症监护病房,’ and ‘quality of life/生活质量.’ This study is supplemented by a manual search of the references of relevant articles and by searching for clinical trial registries.

### 2.5 Inclusion criteria

Studies should meet the following PICO (Population, Intervention, Control, Outcomes) format.

Population: Age ≥ 18 years, staying in the ICU ≥24 hours. Intervention: experimental group receives routine care in the intensive care unit. Meanwhile, medical staff or relatives record the ICU diary (patient's ICU treatment and care experience, e.g. hospitalization scenes, treatment measures, important events, changes in condition, etc.). Control: in the intensive care unit, the control group receives routine care. Outcomes: the QoL score of patients or relatives after ICU (i.e., studies reported at least one of the domains of QoL using validated measurement tools and the tool is not limited).

### 2.6 Exclusion criteria

Exclusion criteria included (1) Review articles and protocol studies. (2) Full‐text not being available. (3) Incomplete and incorrect data are reported in studies. (4) There is no control group. (5) The outcomes indexes are inconsistent.

### 2.7 Search outcomes

**FIGURE 1:**
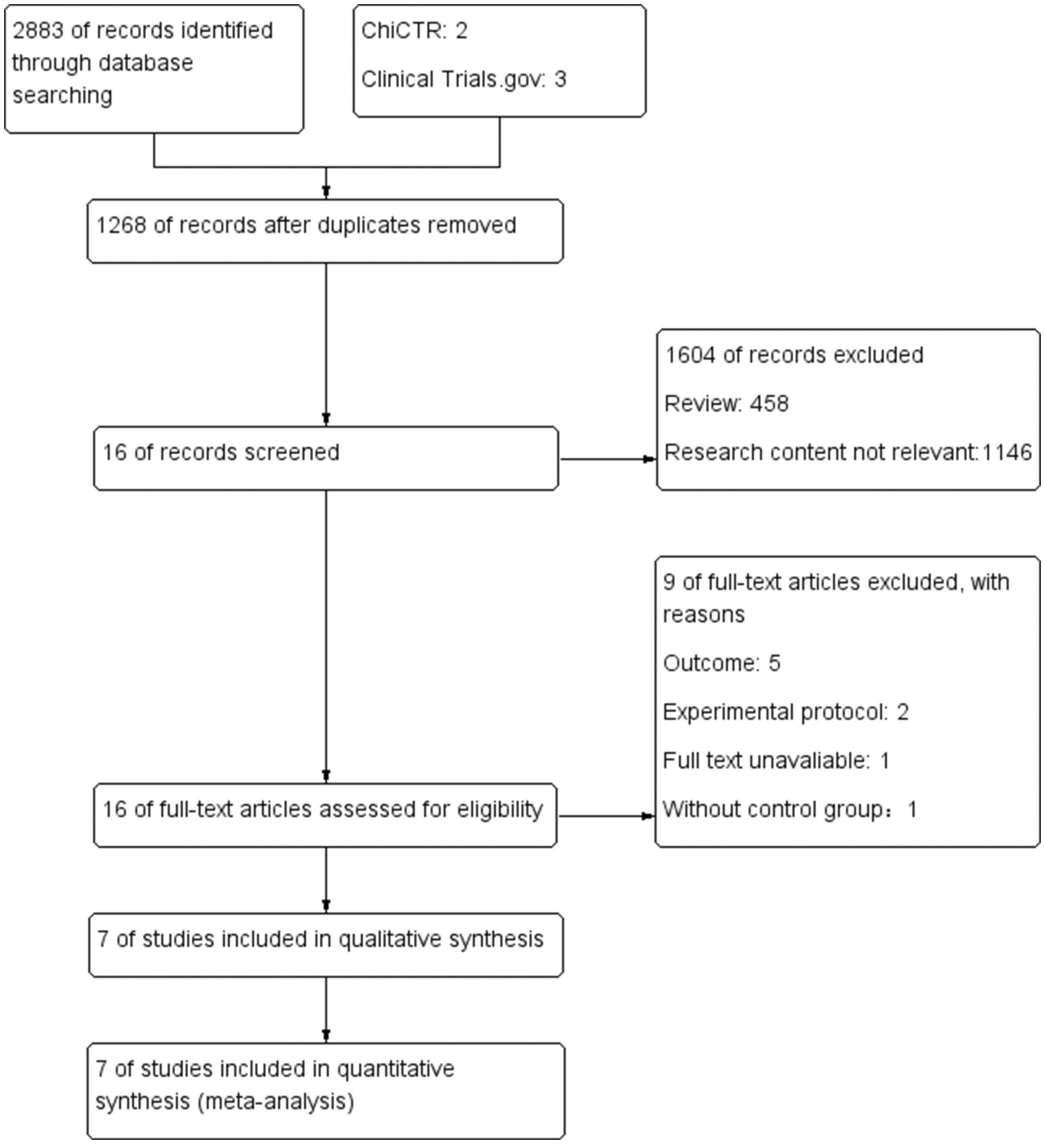
Flowchart of literature search.

We identified 2883 studies that may be related to the subject for screening. Sixteen were selected for a full‐text review of which seven satisfied all inclusion and exclusion criteria. Figure 1 outlines the flowchart of the search. Of those excluded, one had no full text, one had no control group, and in five studies the outcomes included were not the QoL.

### 2.8 Data abstraction

A pre‐defined data extraction form was used for the extraction of data from the included studies. Data collected included author, year, sample size, country, study design, sample source, diary content, diary writer, follow‐up and outcomes. These data were extracted independently by two authors, and disagreements were resolved through a discussion by a third author. We contacted the corresponding author to obtain important data if it was not available in the included studies.

### 2.9 Quality appraisal

Three authors independently used structured tools to assess the methodological quality of the studies included. This included the Cochrane Handbook 5.1.0 for controlled study Bias Risk Assessment Tool (Ghogomu et al., 2014) and the ‘Newcastle–Ottawa Scale’ (NOS) (Stang, 2010). Any disagreement was resolved through a discussion by a fourth author. Cochrane Handbook 5.1.0 has seven assessment items, each of which has ‘low risk,’ ‘unclear’ and ‘high risk.’ The NOS scale includes eight items (9 points), 1 ~ 3 indicating low quality; 4 ~ 6 indicating moderate quality; 7 ~ 9 indicating high quality. It is clear that the ICU diary was not possible to blind medical staff and healthcare providers, but it was possible to blind patients (who were unaware of the study objectives and outcomes) and outcome assessors. Therefore, blinding of patients and outcome assessors is considered adequate.

### 2.10 Synthesis

Data analysis was performed using RevMan 5.3. The Cochrane *Q* test and *I* ^2^ test were performed to assess heterogeneity. If *p* > 0.1, *I* ^2^ < 50%, a fixed‐effect model was used; otherwise a random‐effect model was used. Standardized mean difference (SMD) was used as the measure of effect for continuous outcomes. Calculate the 95% CIs. A *p* value of less than 0.05 was considered significant.

Because of the small number of studies included that could not be pooled, the results of QoL for relatives were presented in qualitative analysis.

In the Cochrane handbook, the particular definition of SMD used in Cochrane Reviews is the effect size known in social science (Higgins et al., 2022). Therefore, 0.2 ~ 0.5 indicates a low effect, 0.5 ~ 0.8 indicates a moderate effect and 0.8 indicates a high effect (Cohen, 1988).

## 3 RESULTS

### 3.1 Study quality

**FIGURE 2:**
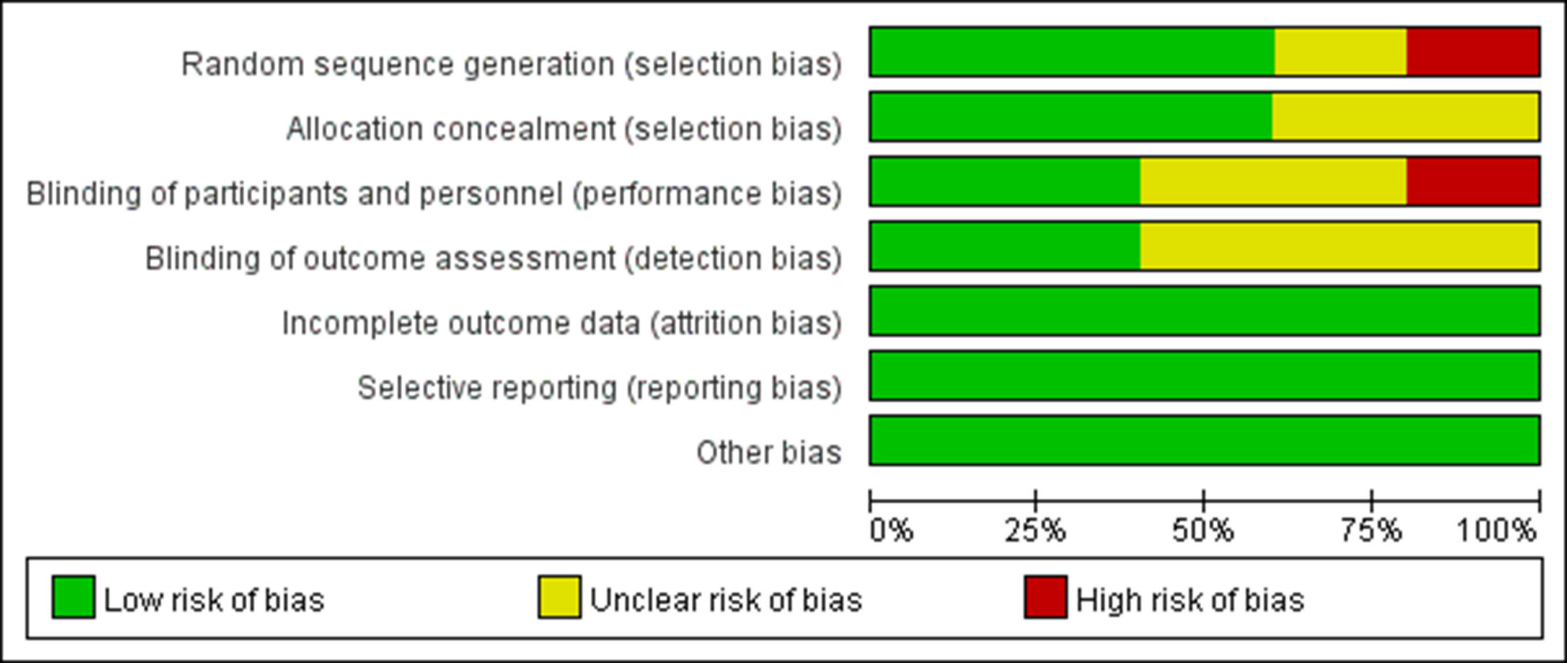
Risk of bias graph for all interventions.

The overall quality of the seven included studies was moderate (Table 1 and Figure 2). Among the five controlled studies, three have described the randomization method. Liu et al. (2019) used a random number table and two studies (Wang, 2017; Wang et al., 2020) used random number website‐aided randomization. Three studies (Nielsen et al., 2019; Wang, 2017; Wang et al., 2020) have mentioned the use of opaque sealed envelopes for allocation concealment. Three studies mentioned blinding for subjects (Wang, 2017; Wang et al., 2020) or outcome assessors (Nielsen et al., 2019; Wang et al., 2020). Also, Wang et al. (2020) mentioned modified intention‐to‐treat analysis. Two studies (Nielsen et al., 2019; Wang, 2017) mentioned reasons for dropout after allocation and excluded them from the analysis. Three studies (Nielsen et al., 2019; Wang, 2017; Wang et al., 2020) mentioned funding and the implementation of outcome measures. Among the two cohort studies, Svenningsen et al. (2014) received five scores out of nine points, and Bäckman et al. (2010) scored seven. There was no disagreement about the risk of bias (Table 1).

**TABLE 1:** Basic characteristics of the included studies.

Author/Year	Country	Study design	Sample size (T/C)	Sample source	Intervention (T/C)	Diary writer	Length of follow‐up	Outcome measured	Quality evaluation
Wang, 2017	China	Randomized controlled trial	43/47	CSICU	Length of stay, treatment process and photos/no diary	Medical staff	3 months	SF‐36	B^a^
Nielsen et al., 2019	Denmark	Randomized controlled trial	26/22	ICU	Events and photos during the patient's ICU/no diary	Relative	3 months	SF‐36	B^a^
Liu et al., 2019	China	Randomized controlled trial	55/55	ICU	Treatment process, mood and photos/no diary	Nurse	3 weeks	QLSBC	B^a^
Wang et al., 2020	China	Randomized controlled trial	41/42	CSICU	Treatment process and photos/no diary	Nurse	3 months	SF‐36	A^a^
Bäckman et al., 2010	Sweden	Cohort studies	38/224	ICU	Photos, diary summary, treatment process/no diary	Medical staff or Relative	6 months	SF‐36	Seven^b^
Svenningsen et al., 2014	Denmark	Cohort studies	43/47	ICU	Length of stay, treatment process and photos/no diary	Medical staff	3 months	SF‐36	Five^b^
Wu, 2020	China	Non‐randomized control	50/50	ICU	Diary/no diary	Nurse and Relative	3 weeks	QLSBC	B^a^

Abbreviations: CSICU, Cardiac surgical intensive care unit; NOS, Newcastle–Ottawa Scale.

^a^ Cochran Manual 5.1.0.

^b^ NOS.

### 3.2 Characteristics of study

Of the seven studies, four were from China, two from Denmark and one from Sweden. The sample size was between 48 and 262 patients. Four of the studies had a follow‐up period of 3 months, while two studies had 3 weeks and one study had 6 months. The measurement of QoL in seven studies was conducted using the 36‐item Short‐Form (SF‐36) and Quality of Life Scale of Breast Cancer (QLSBC). Four studies were written on the ICU diaries by medical staff, two were written by medical staff or relatives, and one was the nursing taught the relatives' writing of the diary. Six studies used photos when writing the ICU diaries (Table 1). The content of the diary was mainly displayed to the patient after discharged from the hospital for the purpose of informing them of an event that occurred during the ICU and the diary was handed over to them (Bäckman et al., 2010; Nielsen et al., 2019; Wang, 2017; Wang et al., 2020) at the end of the follow‐up.

### 3.3 Outcome analysis

Seven studies reported total scores or domains for the QoL separately. Furthermore, some outcomes included a small number of studies and could not be pooled. Therefore, we performed a meta‐analysis of overall QoL and its domains. In addition, we also performed a qualitative analysis of QoL in relatives.

#### 3.3.1 Total QoL

**FIGURE 3:**
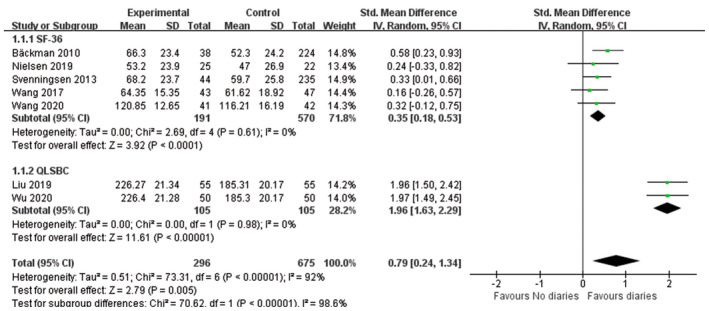
Forest plot of effects of ICU diaries on overall quality of life for patients.

Seven studies reported on different combinations of domains of the QoL, including 971 participants. We performed the meta‐analysis on overall QoL, there was a statistically significant improvement in QoL with diaries, with a pooled SMD 0.79 (moderate effect) (95% CI, 0.24–1.34; *p* = 0.005). However, there was a high heterogeneity in the combined studies. Sensitivity analysis demonstrated that the results of the fixed effects model change were stable (SMD = 0.71, moderate effect, 95% CI:0.55–0.87, *p* < 0.001). Similarly, the subgroup analysis according to the QoL scale also found that ICU diaries can improve the QoL of patients (Figure 3). To explore the specific role of ICU diaries on QoL, we performed a subgroup analysis according to the components of SF‐36 and QLSBC.

#### 3.3.2 Sf‐36

**FIGURE 4:**
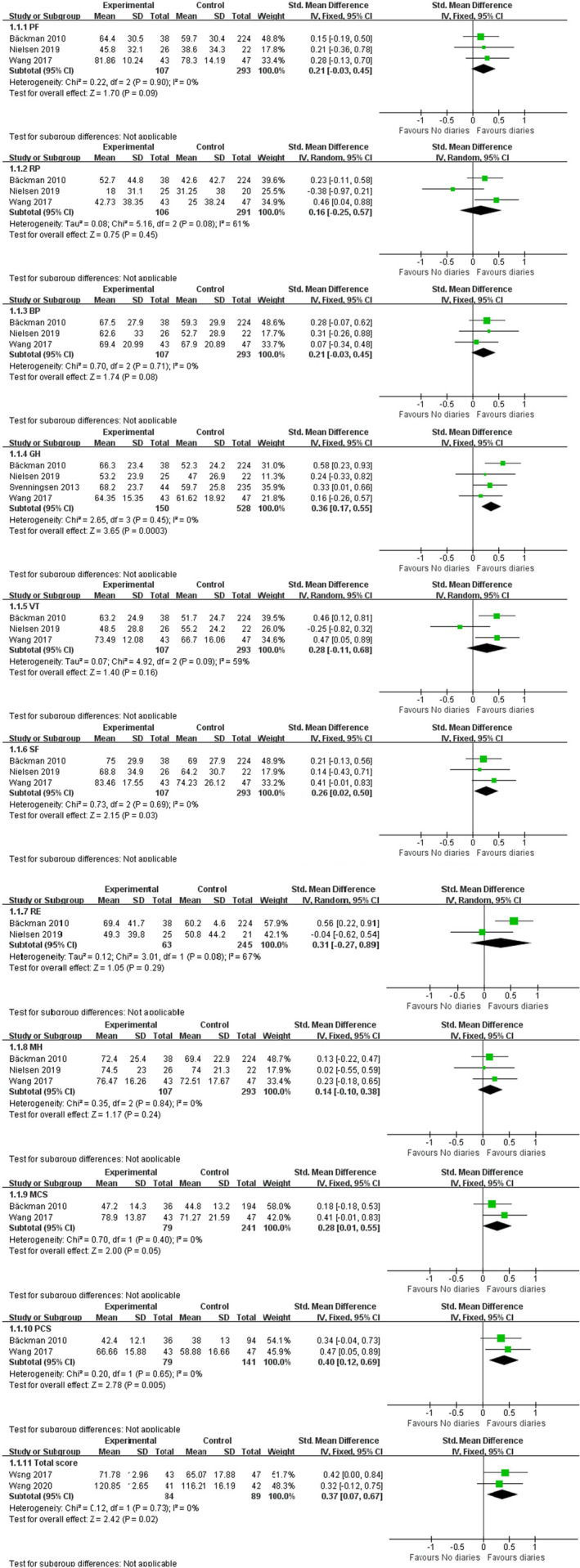
Forest plot of effects of the ICU diaries on 11 components of SF‐36 for patients.

Five studies reported on different combinations of domains of the SF‐36. The result of the meta‐analysis showed that compared with the control group, ICU diaries can significantly improve Global health (SMD = 0.36, low effect, 95% CI [0.17, 0.55], *p* = 0.0003), Social function (SMD = 0.26, low effect, 95% CI [0.02, 0.50], *p* = 0.03), Physical component summary (SMD = 0.40, low effect, 95% CI [0.12, 0.69], *p* = 0.005) and Total score (SMD = 0.37, low effect, 95% CI [0.07, 0.67], *p* = 0.02) for patients. The remaining domains of the SF‐36 showed no difference between the ICU diaries and the control group (Figure 4).

#### 3.3.3 QLSBC

Two studies reported on different combinations of domains of the QLSBC. The result of the meta‐analysis showed that compared with the control group, ICU diaries can significantly improve patients’ Physical Well‐Being (SMD = 1.21, high effect, 95% CI [0.92, 1.51], *p* < 0.001), Psychological Well‐Being (SMD = 1.18, high effect, 95% CI [0.89, 1.47], *p* < 0.001), Social Well‐Being (SMD = 0.43, low effect, 95% CI [0.15, 0.70], *p* = 0.002), Spiritual Well‐Being (SMD = 0.50, low effect, 95% CI [0.22, 0.77], *p* = 0.0004) and Total score (SMD = 1.96, high effect, 95% CI [1.63, 2.29], *p* < 0.001).

#### 3.3.4 Relatives

Only Nielsen et al. (2019) evaluated the impact of ICU diaries on relatives' QoL using the SF‐36. The results showed that relatives who received ICU diaries had higher mean scores for the domains of the SF‐36 at 90 days after ICU discharge, but the difference in higher scores was not statistically significant (*p* > 0.05).

## 4 DISCUSSION

Overall, our study provides a re‐evaluation of the effect of ICU diaries on patient QoL. Although some studies reported significant findings, they did not provide the data needed to analyse the merger. Based on the above limits and current research progress, we found a beneficial effect of ICU diaries on survivors' QoL (overall: SMD = 0.79). Similar findings were found for subgroup analysis based on differences in the questionnaire. For the SF‐36 domains, our study showed a low effect of ICU diaries in improving GH (SMD = 0.36) and SF (SMD = 0.26), and showed only a positive trend for the other domains. Our study showed a high effect of ICU diaries in improving the four QLSBC domains. But the efficacy of ICU diaries on relatives' QoL was not found.

Studies of diaries as non‐pharmacological interventions for PICS are gaining attention. Recent studies have shown that ICU diaries help facilitate the reproduction and reconstruction of patients' memories and improve their prognosis and QoL (Ullman et al., 2015; Veloso Costa et al., 2021). Two recent meta‐analysis results reported improvement in the QoL‐GH of ICU survivors (Barreto et al., 2019; McIlroy et al., 2019) and another meta‐analysis conducted a descriptive analysis of QoL. Based on this, we conducted a meta‐analysis that summarized these findings suggesting a benefit with ICU diaries, including four new trials. We suppose that the difference between our study and the meta‐analysis may be explained by the difference in evaluated tools and sample source. Although there was heterogeneity, the results were stable and more reliable.

The effect of diaries on the SF‐36 domains is controversial. An earlier study reported an improvement in each SF‐36 domain except MH and GH for more than 1–12 months of follow‐up (Dowdy et al., 2005), but a recent study found improvements in HRQOL in the first year after discharge were mainly seen for PF, RP, VT and SF (Gerth et al., 2019). When screening the literature, in the Pattison et al. (2019) study, patients who received a diary had a lower mean score for the EuroQol at 12 months compared to 4 months. Bäckman et al. (2010) found an improvement in the GH and VT after controlling for factors influencing the QoL. When pooling the trials, we found an improvement in GH and SF, and this reflects the efficacy of diaries. The length of stay in the hospital and greater illness severity may be associated with reduced QoL. Reading diaries may add to feelings of guilt, leading to psychological harm (Pattison et al., 2019). This may explain the insignificant effect of the ICU diary on the domains of SF‐36. We found that the ICU diary only improved PCS, Stricker et al. (2005) found no differences in the PCS and MCS of the length of stay in the ICU, which is different from Langerud et al. (2018) who reported an improvement only in PCS from 3 months to 1 year after discharge. It means that the ICU diary has a large effect. Before discharge from the ICU, a number of patients had severely impaired physical or more severely ill on admission, so survival is clearly an important outcome, but life after critical illness is a second outcome, and therefore mental QoL may be neglected. This is consistent with Maslow's theory, which focused more on physical symptoms and neglected psychological issues. With the use of a diary, we believe that the expected mental health will be achieved. However, ICU survivors have lower QoL than the normative population at 6 months following ICU discharge (Jackson et al., 2009).

It is clear that not all patients have the same needs and some may actively recall and reconstruct their stories from their time in the ICU; others may just want to move on. The ICU diary is a double‐edged sword. On the one hand, reading diaries can help survivors understand how slowly they are recovering and increase their making sense of the ICU experience. 44% of patients read their diaries multiple times and 60% found them easy to understand and full of memories (Egerod & Bagger, 2010; Glimelius Petersson et al., 2018). Nielsen et al. (2019) reported that 95% of patients had discussed the diary with their relatives. Reading the diary feel supported by relatives and increase family cohesion, and may have positive effects. On the other hand, the psychological recovery of survivors after discharge is very slow. Reading a diary can relieve the ICU experience, evoke memories, and make patients feel scared (Johansson et al., 2015). O'Gara and Pattison (2016) reported that some patients were reluctant to discuss the diary with others. 20% of patients or relatives did not want a diary (Halm, 2019). Wang et al. (2020) found that patients refused to read the ICU diary or did not like talking about their ICU experiences. Providing sensitive or private information in deciding which patients benefit from diaries may lead to unintended psychological harm, dilute the effects of the diary and delay recovery and return to society. Therefore, when implementing diaries, there is a need to target the right people to gain the most from diaries.

Because of the differences in location, culture and medical settings, there is no common standard framework for diaries, and the content of diaries varies from person to person. In the future, diaries need to target the right people and describe the entire process in detail for replication and implementation; hospitals need to rationalize their workload to avoid burdening staff with diaries and form relevant laws to protect the use of diaries. Finally, an Internet for address of ICU diary bases (http://www.icu‐diary.org/diary/start.html) provides evidence of diaries.

Limitations: Seven studies reported significant findings, but some did not provide sufficient information needed for meta‐analysis, so we only meta‐analysed two or three studies, with a high risk of bias and inaccurate results, which is a major limitation. The influence of study design, follow‐up times and evaluation tools, which may increase the heterogeneity across studies. We only included studies in Chinese or English. Studies in other languages may also report the information required for this study, which may lead to publication bias. A small sample size and over half of the included studies had patients or relatives who were not interested in diaries and refused visits. Bäckman et al.'s (2010) had a 48% rate of loss to follow‐up, which may interfere with the efficacy of the diary intervention and hinder the generalization of the results. Only one study described the blinding of patients and outcome assessors, therefore reducing the reliability of results and the effect of the diary. In addition, only one quantitative study reported diaries do not have benefits for relatives' QOL, but diaries may be helpful for family members who experience the loss of a loved one, according to a systematic review (Galazzi et al., 2022). These results should be verified further by more quantitative studies.

## 5 CONCLUSION

In summary, this study highlighted the efficacy of ICU diaries in improving ICU survivors' overall QoL as well as domains of GH, SF and four domains of QLSBC, but the QoL of their relatives did not improve. In the future, more content will be needed to continue developing, such as legal, hospital and a common framework of diaries, and to provide a basis for further ICU diary follow‐up clinics.

## AUTHOR CONTRIBUTIONS

DH conceived the study and wrote the article. DH and YL conducted a literature search. DH, YL and XJ conducted a data collection. JC, DH, YL, XJ and YL revised the article. All authors read and approved the final manuscript.

## FUNDING INFORMATION

This research did not receive any specific grant from funding agencies in the public, commercial, or not‐for‐profit sectors. The research protocol has been registered in the PROSPERO system evaluation registration (CRD42021251916).

## CONFLICT OF INTEREST STATEMENT

The authors declare no conflicts of interest.

## Data Availability

The data that support the findings of this study are available from the corresponding author upon reasonable request.
